# Evaluating Percutaneous Gallbladder Drainage: 10-Year Outcomes From a Large District General Hospital in the UK

**DOI:** 10.7759/cureus.113410

**Published:** 2026-07-26

**Authors:** Ahmed Abdelwahed, Dadhakrishnan Ganesh, Stephen McNally

**Affiliations:** 1 Upper GI and General Surgery, Raigmore Hospital NHS Highland, Inverness, GBR

**Keywords:** acute cholecystitis, endoscopic ultrasound-guided gallbladder drainage (eugbd), gallbladder drainage, high-risk patients., percutaneous transhepatic gallbladder drainage (ptgbd)

## Abstract

Background

Acute cholecystitis (AC) is a common surgical emergency, and early laparoscopic cholecystectomy (LC) is considered the standard treatment. However, some patients are unsuitable for surgery due to advanced age, multiple comorbidities, or poor physiological reserve. In these cases, gallbladder drainage is required, most commonly performed using percutaneous transhepatic gallbladder drainage (PTGBD).

This study evaluates the outcome of PTGBD in a large UK District General Hospital (DGH).

Methods

A retrospective analysis was performed of all patients undergoing PTGBD between November 2013 and December 2023 in a single UK DGH. Data collected included demographics, indications, procedural success, complications, need for reintervention, mortality, and subsequent surgical management.

Results

A total of 47 patients underwent PTGBD (55.3% male, n=26), 71.7% American Society of Anesthesiologists (ASA) grade 3 (n=34). The main indications were AC (78.7%, n=37) and gallbladder perforation (19.1%, n=9). Technical and clinical success rates were 97.8% (n=46) and 92.3% (n=43), respectively. Thirty-day and 90-day mortality were 2.2% (n=1) and 6.8% (n=3), respectively. Reintervention was required in 43.5% of patients (n=20), and 54% (n=25) experienced unplanned readmission, most commonly due to recurrent cholecystitis or drain-related complications.

Conclusion

PTGBD is an effective, early source control in high-risk patients with AC, with high success rates and acceptable mortality. However, high rates of reintervention and readmission highlight important limitations of this approach. Its use should depend on local expertise and careful outcome monitoring.

## Introduction

Acute cholecystitis (AC) is a common cause of emergency surgical admission and represents a significant clinical and economic burden on healthcare systems. It usually occurs due to obstruction of the cystic duct by gallstones, leading to gallbladder inflammation, infection, and, in severe cases, necrosis or perforation [1-3].

Laparoscopic cholecystectomy (LC) is considered the standard treatment for AC and is recommended early in the disease course to reduce recurrence, complications, and overall healthcare use [3-5]. This was also recommended by the international consensus guidelines, including the Tokyo Guidelines 2018, which recommend early surgical intervention in the form of LC where feasible, even in moderate to severe cases, provided appropriate expertise is available [1,2,6]. However, a substantial proportion of patients presenting with AC are elderly or have significant comorbidities, such as cardiovascular, respiratory, or renal disease, making them high-risk surgical candidates. In these patients, emergency cholecystectomy is associated with higher perioperative morbidity and mortality, so alternative strategies are often required to achieve source control [3,7].

Percutaneous transhepatic gallbladder drainage (PTGBD) is commonly used in high-risk patients, offering high technical success rates and rapid clinical improvement [8]. The procedure involves placing a catheter into the gallbladder under image guidance, allowing decompression of the inflamed gallbladder and effective control of sepsis. High technical success rates were reported, with clinical success seen in most patients within the first few days following intervention [9-11].

In addition to being effective, PTGBD is widely available and can be performed under local anesthesia with ultrasound or CT guidance, making it particularly suitable for patients who are not fit for surgery. It is also the preferred option for patients who are expected to undergo surgery at a later date, as it is used as a bridge to delayed cholecystectomy [12], and it can offer definitive management in selected individuals. [7-9,13]. Despite these advantages, PTGBD has several limitations. The use of external drainage catheters can affect the quality of life of the patients and cause complications such as dislodgement, blockage, bile leakage, and infection. It also does not treat the underlying gallstone disease, so patients remain at risk of recurrent biliary events and repeated hospital admissions [9,14].

PTGBD may be used either as a bridge to delayed cholecystectomy in patients who become suitable for surgery following recovery, or as a definitive treatment in patients who remain unsuitable for operative intervention because of persistent comorbidity or frailty.

This study aims to assess the outcomes of PTGBD over a 10-year period in a large UK District General Hospital (DGH), focusing on procedural success, complications, reintervention, and patient outcomes, including its role as both a bridge to delayed cholecystectomy and as a definitive treatment in high-risk patients.

## Materials and methods

Study design

This was a retrospective observational cohort study conducted at a large DGH in the UK over a 10-year period (November 2013 to December 2023) to assess outcomes of PTGBD. The hospital serves both urban and rural populations and offers emergency general surgical and interventional radiology services. 

Ethical approval

Ethical approval was not required because the study was retrospective and used anonymised patient data. The study followed the local data protection regulations. No patient-identifiable information was recorded. 

Patient selection

All consecutive patients who underwent PTGBD during the study period were identified from the radiology information system and checked against electronic patient records. The inclusion and exclusion criteria are summarised in Table 1. 

**Table 1:** Inclusion and exclusion criteria. PTGBD, percutaneous transhepatic gallbladder drainage.

Inclusion criteria	Exclusion criteria
Patients who underwent PTGBD for suspected or confirmed gallbladder disease	Patients with incomplete clinical records
Radiologically or clinically diagnosed acute cholecystitis	Procedures performed for non-biliary indications
Patients deemed unfit for immediate surgical intervention	Patients who underwent alternative gallbladder drainage techniques

Patient suitability for PTGBD was assessed by a multidisciplinary team including surgeons and interventional radiologists based on clinical assessment and patient comorbidities.

Patients were considered for PTGBD when they were deemed unsuitable for emergency LC because of significant physiological risk, advanced age, severe comorbidities, sepsis requiring urgent source control, or unfavourable operative risk following multidisciplinary discussion between emergency surgery and an interventional radiologist.

Diagnosis and severity assessment

The diagnosis of AC was based on a combination of clinical, biochemical, and radiological findings following standard criteria. This included right upper quadrant pain and tenderness, raised inflammatory markers (e.g., white cell count, C-reactive protein), and radiological evidence of gallbladder inflammation on ultrasound or CT. Severity was determined from clinical presentation and physiological status. Formal Tokyo Guidelines 2018 severity grades were not consistently documented because of the retrospective nature of the study and therefore were not analysed. Patients' comorbidities and American Society of Anesthesiologists (ASA) status were captured. 

Procedure technique

All PTGBD procedures were performed by experienced interventional radiologists under ultrasound and/or CT guidance. Procedures were usually performed under local anaesthesia. Correct catheter placement was confirmed radiologically, and bile drainage was established. Catheters were secured externally and connected to drainage bags.

Post-procedure care and follow-up

Following PTGBD, patients were monitored for clinical improvement and resolution of sepsis symptoms. Blood tests, including inflammatory markers and liver function tests, were checked regularly. Drain management included monitoring drain output volume and colour, flushing as per local protocol, and imaging (e.g., cholangiography) where clinically indicated. Decisions regarding drain removal, replacement, or further intervention were made based on clinical progress and team discussions. Patients were followed up through electronic records to identify reinterventions, complications, readmissions, and interval cholecystectomy. Drain removal was considered after clinical resolution of sepsis together with satisfactory imaging or cholangiographic findings demonstrating cystic duct patency where appropriate. Interval cholecystectomy was offered following multidisciplinary review when the patient became medically fit for surgery.

Outcome measures

Primary outcomes included technical success, which was identified as successful placement of a catheter in the gallbladder with immediate bile drainage. Clinical success was defined as resolution of sepsis and improvement in symptoms without the need for urgent further intervention. Secondary outcomes included 30-day and 90-day mortality, reintervention within 30 days, unplanned readmissions, drain-related complications, and definitive management (interval cholecystectomy). 

Reintervention was defined as any additional radiological or surgical procedure related to gallbladder disease within 30 days of the index procedure. Unplanned readmission was defined as any emergency hospital admission following discharge related to biliary disease or drain-related complications. Complications were defined as catheter dislodgement, blockage, infection, bile leak, or requirement for repeat drainage.

Data collection and management

Data were extracted from electronic patient records, radiology systems, and operative databases. All data were anonymised and stored securely as per hospital data governance policies. Collected variables included patient demographics (age, sex), ASA grade, indication for PTGBD, procedure details, and clinical outcomes, including complications. Data completeness was assessed during data extraction. Patients with incomplete clinical records were excluded according to the predefined exclusion criteria, and analyses were performed using the available data only.

Statistical analysis

Descriptive statistical analysis was performed. Continuous variables were summarised using mean or median where appropriate, and categorical variables were expressed as percentages. Given the observational design and relatively small sample size, no comparative statistical testing was performed. 

## Results

Patient demographics

A total of 47 patients underwent PTGBD during the study period. The cohort showed a slight male predominance, with 55.3% male (n=26) and 44.7% (n=21) female patients. Most patients were considered high-risk surgical candidates, with 71.7% (n=34) having an ASA grade of 3 and 6.4% (n=3) ASA grade of 4. This represents a group of patients with significant comorbidities and limited suitability for surgery. The median age of this group was 79 years. 

Indications for PTGBD

**Figure 1:**
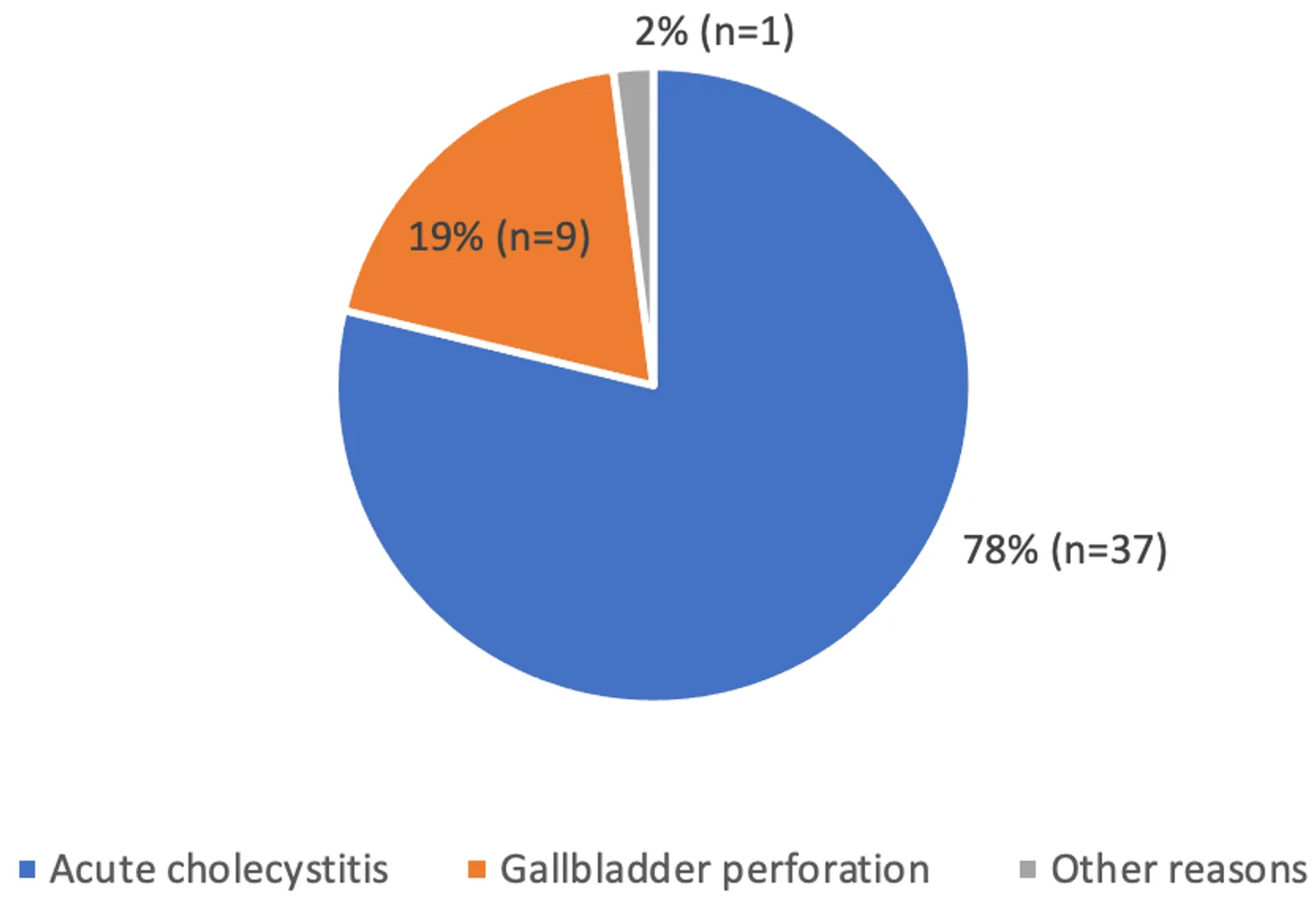
Indications for PTGBD, showing acute cholecystitis as the main indication. PTGBD, percutaneous transhepatic gallbladder drainage.

The primary indication for PTGBD was AC, seen in 78.7% of cases (n=37). Gallbladder perforation was the second most common indication, seen in 19.1% of patients (n=9). A small minority of patients (2.2%, n=1) underwent PTGBD for other reasons. The indications of PTGBD are summarised in Figure 1. 

Procedural outcomes

**Figure 2:**
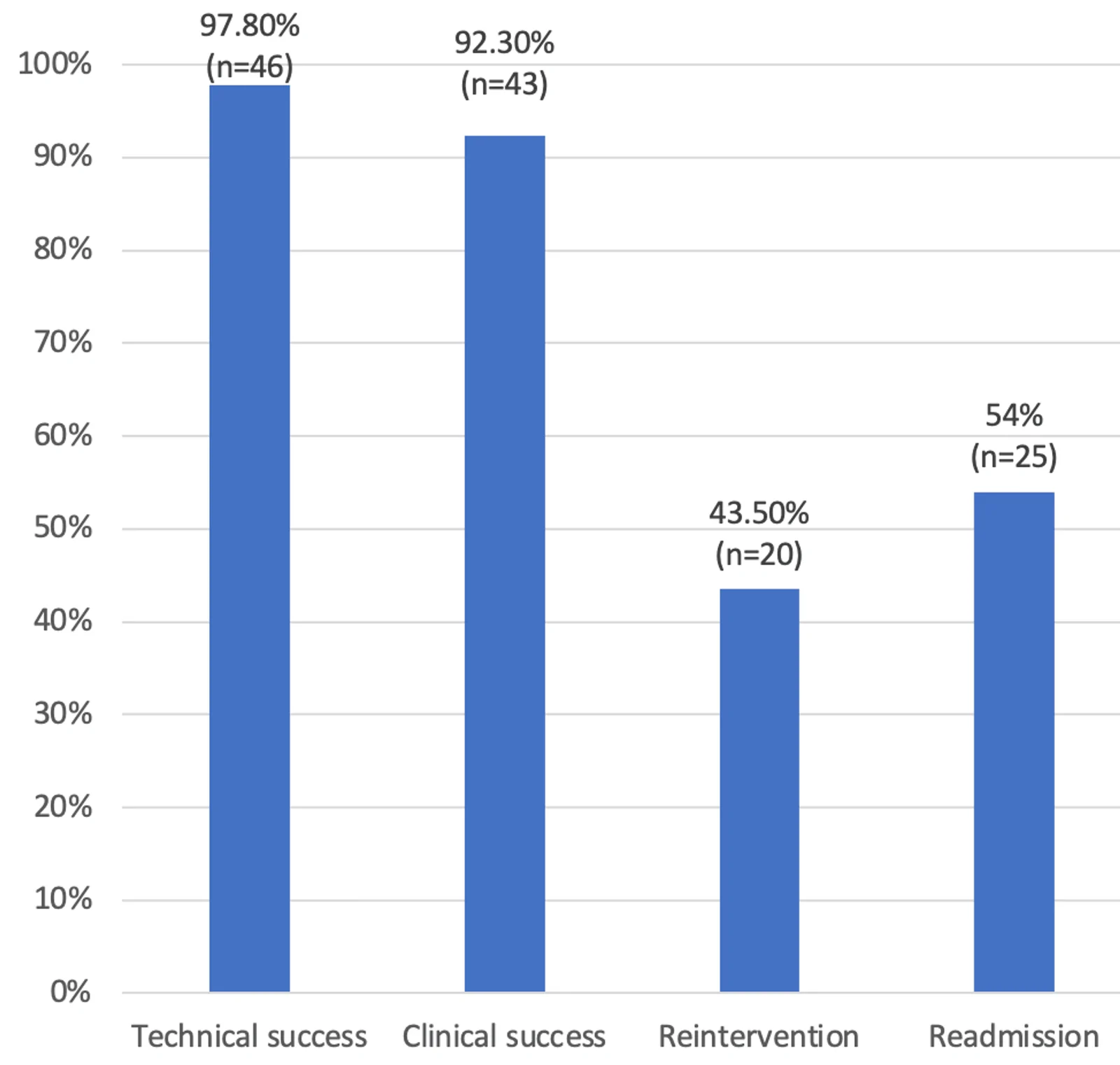
Summary of outcomes after PTGBD. PTGBD, percutaneous transhepatic gallbladder drainage.

PTGBD demonstrated a technical success rate of 97.8% (n=46), with successful catheter placement in almost all cases. Clinical success, defined as resolution of sepsis and improvement in symptoms without urgent further intervention, was achieved in 92.3% (n=43) of patients. The procedural outcomes are summarised in Figure 2. 

Mortality 

**Figure 3:**
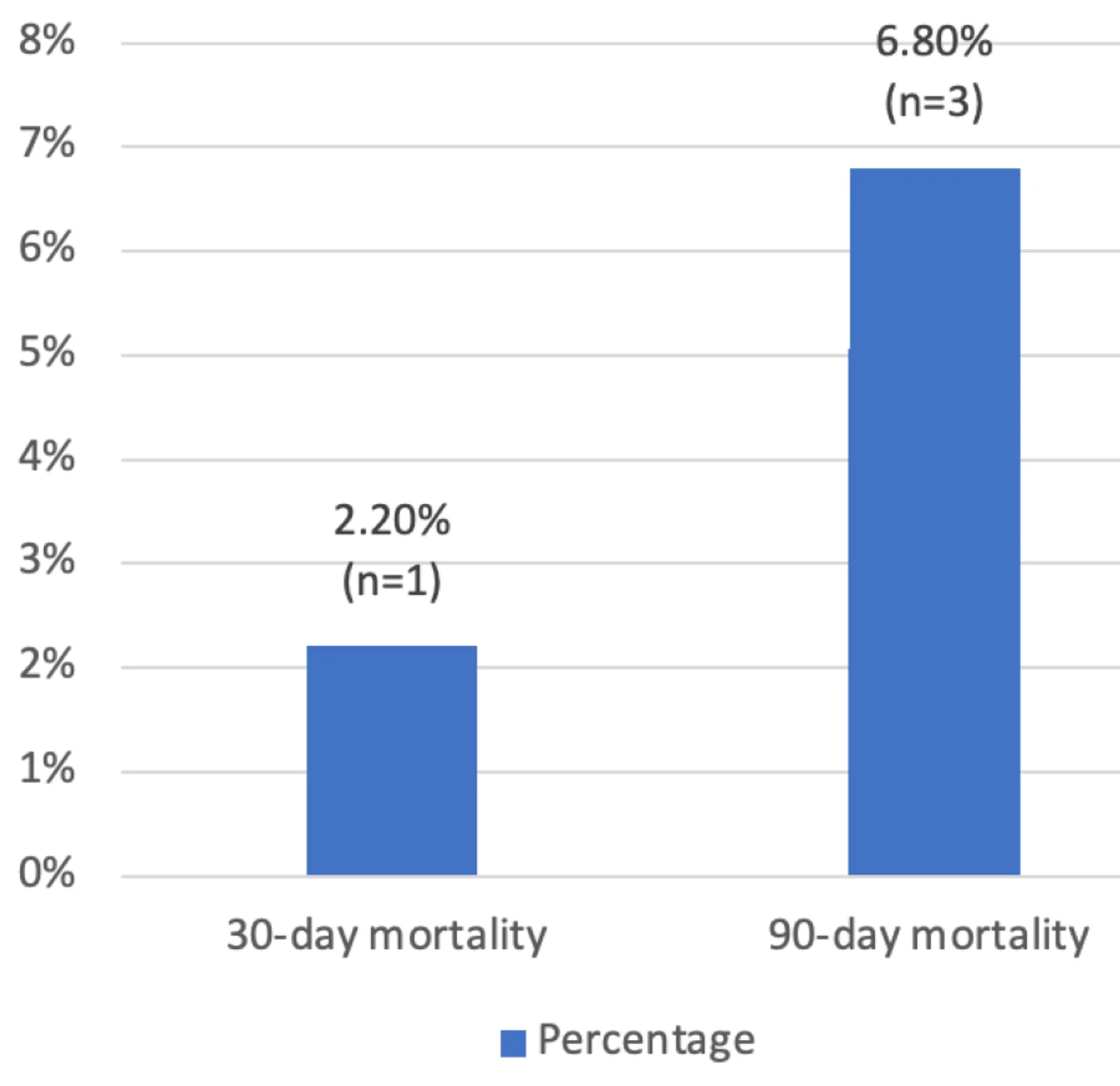
Mortality rates at 30 and 90 days after PTGBD. PTGBD, percutaneous transhepatic gallbladder drainage.

Thirty-day mortality was 2.2% (n=1), and 90-day mortality was 6.8% (n=3). No death was related to the procedure. Mortality outcomes are shown in Figure 3. 

Reintervention

A number of patients required further intervention after initial PTGBD. The reintervention rate within 30 days was 43.5% (n=20). The most common reintervention was cholangiography, seen in 50% of cases (n=10), mainly to check biliary anatomy or catheter function. Catheter replacement was needed in 10% of patients (n=2), usually due to blockage or displacement. Other interventions, including further drainage procedures, were performed in 10% of patients (n=2). The types of reinterventions are shown in Table 2. 

**Table 2:** Reintervention

Procedure	Percentage (number)
Cholangiogram	50% (n=10)
Catheter replacement	10% (n=2)
Further drainage	10% (n=2)

Readmissions

**Figure 4:**
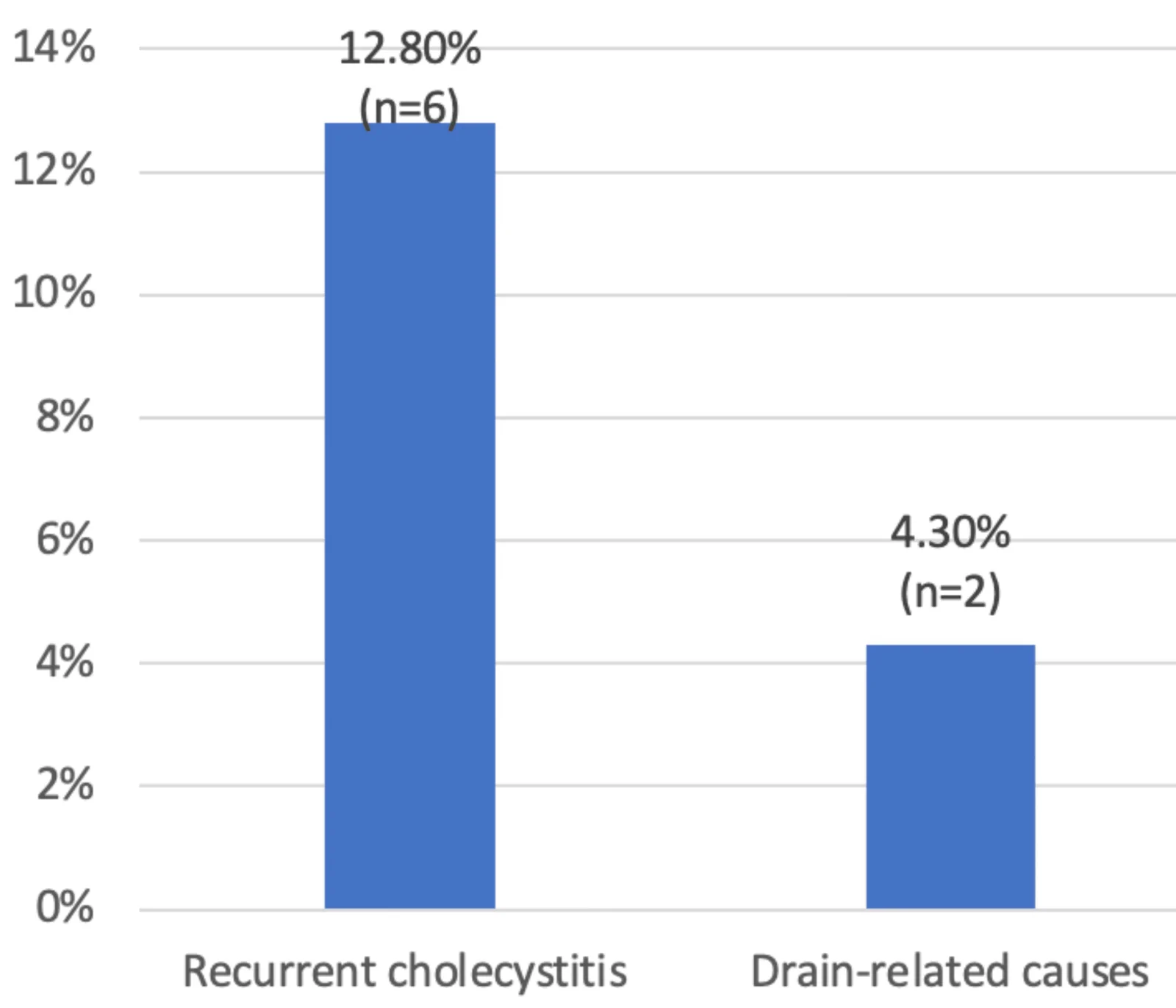
Causes of unplanned readmission following PTGBD, highlighting recurrent cholecystitis as the most common reason. PTGBD, percutaneous transhepatic gallbladder drainage.

Unplanned hospital readmissions were common, with 54% (n=25) of patients requiring at least one readmission following discharge. The most frequent cause of readmission was recurrent cholecystitis occurring in 12.8% (n=6). Other causes included drain-related complications such as tube dislodgement or accidental removal, occurring in 4.3% (n=2). The causes of readmissions are shown in Figure 4. 

Definitive surgical management

A number of patients subsequently underwent cholecystectomy following initial stabilisation. Seven patients (14.9%) underwent interval cholecystectomy after recovery from the acute episode, while 18 patients (38.3%) underwent cholecystectomy at a later stage. Overall, PTGBD acted as a bridge to surgery in almost half of the patients (25/47, 53.2%). However, a significant proportion remained unsuitable for surgery and required ongoing drain management (22/47, 46.8%). 

## Discussion

This study shows that PTGBD is an effective intervention for early source control in high-risk patients with AC. The technical and clinical success rates in this study are 97.8% and 92.3%, respectively, which are similar to previously reported success rates of 90-100% [9,11,15,16]. The high clinical success seen reflects the ability of PTGBD to quickly control sepsis and stabilise patients who would otherwise be at significant risk of deterioration if managed conservatively or undergo emergency surgery. In this predominantly ASA 3 population, these findings support the use of PTGBD in frail and comorbid patients, where surgery carries substantial risks [7-9]. The median age of 79 years further reflects that PTGBD was primarily used in an elderly, frail population in whom emergency cholecystectomy would have carried substantial operative risks. 

Our findings are consistent with the published literature demonstrating that PTGBD is a highly effective treatment for AC in patients who are unsuitable for surgery. The technical success rate of 97.8% and clinical success rate of 92.3% observed in our cohort are compatible with those reported in systematic reviews and meta-analyses, where technical success typically exceeds 95% and clinical success ranges from approximately 87% to over 90% [17,18]. Similarly, the low mortality observed in our study reflects previous reports, suggesting that mortality following PTGBD is largely related to advanced age, comorbidities, and severity of AC rather than the procedure itself [17,19].

PTGBD is a relatively safe and less invasive treatment option. The reported mortality related to the procedure is 0-4% [9]. The low mortality rates seen in this study, with 30-day mortality of 2.2% and 90-day mortality of 6.8%, support the safety of PTGBD, particularly given the high-risk nature of the patient population. These findings suggest that mortality is more likely related to underlying comorbidities and disease severity rather than the intervention itself, as reported in previous studies [7-9]. Importantly, over half of the patients in this study proceeded to interval or definitive cholecystectomy, supporting the use of PTGBD as a bridge to surgery, allowing time for optimisation of comorbidities and recovery from acute inflammation prior to definitive surgical management [7,9,15]. PTGBD therefore provides both early source control and an opportunity for staged definitive surgery.

However, despite these favourable short-term outcomes, the reintervention rate of 43.5% and readmission rate of 54% highlight the ongoing burden of PTGBD. These findings may be due to the limitations of external drains, including catheter-related complications and ongoing gallstone disease [7,9,20]. The higher readmission rate, most commonly due to recurrent cholecystitis, reinforces that, while PTGBD is effective in controlling the initial episode, it does not represent definitive treatment. This is particularly relevant in patients who are not candidates for subsequent cholecystectomy, where long-term reliance on drainage may result in dislodgement, bile leakage, repeated hospital admissions, and reduced quality of life, together with a requirement for ongoing drain care and follow-up [15,20].

This study has several limitations that should be acknowledged. This was a retrospective single-centre study conducted in a DGH, and the findings may not be applicable to centres with different patient populations. In addition, the sample size was relatively small, and no comparative statistical analysis was performed. There is also a possibility of selection bias, although patients selected for PTGBD were identified by multidisciplinary clinical judgement and were generally high-risk surgical candidates. Despite these limitations, the study provides useful real-world data on the outcome of PTGBD in a high-risk patient population within a DGH setting. Furthermore, formal Tokyo Guideline severity grading was not consistently documented because of the retrospective nature of the study and therefore could not be incorporated into the analysis. In addition, management strategies, imaging availability, and peri-procedural care may have evolved over the 10-year study period, potentially influencing outcomes.

Our finding of high readmissions aligns with other studies demonstrating a high re-presentation rate of over 50% of PTGBD patients [21]. This highlights the need to explore alternative drainage approaches such as endoscopic ultrasound gallbladder drainage (EUGBD). Although EUGBD was not evaluated in this study and no direct comparison with PTGBD was undertaken, it is an evolving alternative. 

EUGBD is a minimally invasive technique using a lumen-apposing metal stent (LAMS) to create internal drainage between the gallbladder and the gastrointestinal lumen, placed under endoscopic ultrasound (EUS) guidance [22,23]. EUGBD offers similar technical and clinical success rates to PTGBD, with lower rates of complications, reintervention, and hospital admissions [12,24-28]. Furthermore, EUGBD permits definitive management of gallstones through endoscopic stone clearance from the gallbladder via the LAMS, reducing recurrent biliary events [29,30].

Comparative studies show that, while PTGBD achieves excellent technical and clinical success, EUGBD is associated with lower rates of recurrent cholecystitis, complications, reintervention, hospital readmission, and shorter hospital stay, supporting its use in centres with appropriate expertise [17,24,31]. Our findings of high rates of reintervention and unplanned readmission associated with PTGBD could suggest that implementation of EUGBD could be considered in order to decrease overall healthcare utilisation.

Currently, despite its potential advantages, EUGBD access remains limited to larger centres, as it requires advanced endoscopic expertise [30]. Consequently, PTGBD remains the primary treatment modality in many centres [32,33]. Furthermore, PTGBD remains a safe, effective, and widely accessible first-line intervention that can be performed under local anaesthesia, with minimal resources, and in patients with unfavourable anatomy or gastrointestinal disease which might preclude EUGBD [9,15,20,34,35].

Therefore, PTGBD remains an important treatment for high-risk patients. However, a tailored, centre-specific approach is required, balancing availability and reliability of PTGBD with the potential of EUGBD to improve patient outcomes. 

## Conclusions

PTGBD is a safe and effective option for managing AC in high-risk patients, providing good early source control with good outcomes. However, high rates of reintervention and readmission highlight its limitations.

Although not evaluated in this study, EUGBD may offer advantages in selected centres with appropriate expertise. Prospective multicentre studies comparing PTGBD and EUGBD with large patient cohorts and standardised outcome measures are required to define the optimal management strategy for non-surgical high-risk patients with AC.
